# Inactivation of the Mouse L-Proline Transporter PROT Alters Glutamatergic Synapse Biochemistry and Perturbs Behaviors Required to Respond to Environmental Changes

**DOI:** 10.3389/fnmol.2018.00279

**Published:** 2018-08-20

**Authors:** Daniel Schulz, Julia Morschel, Stefanie Schuster, Volker Eulenburg, Jesús Gomeza

**Affiliations:** ^1^Institute for Pharmaceutical Biology, University of Bonn Bonn, Germany; ^2^Institute of Biochemistry, University of Erlangen-Nuremberg Erlangen, Germany; ^3^Department of Anesthesiology and Intensive Care Medicine, University of Leipzig Leipzig, Germany

**Keywords:** neurotransmitter transporter, L-proline transporter, PROT, knockout mice, approach-avoidance

## Abstract

The endogenous neutral amino acid L-proline exhibits a variety of physiological and behavioral actions in the nervous system, highlighting the importance of accurately regulating its extracellular abundance. The L-proline transporter PROT (*Slc6A7*) is believed to control the spatial and temporal distribution of L-proline at glutamatergic synapses by rapid uptake of this amino acid into presynaptic terminals. Despite the importance of members of the *Slc6* transporter family regulating neurotransmitter signaling and homeostasis in brain, evidence that PROT dysfunction supports risk for mental illness is lacking. Here we report the disruption of the PROT gene by homologous recombination. Mice defective in PROT displayed altered expression of glutamate transmission-related synaptic proteins in cortex and thalamus. PROT deficiency perturbed mouse behavior, such as reduced locomotor activity, decreased approach motivation and impaired memory extinction. Thus, our study demonstrates that PROT regulates behaviors that are needed to respond to environmental changes *in vivo* and suggests that PROT dysfunctions might contribute to mental disorders showing altered response choice following task contingency changes.

## Introduction

The amino acid L-proline is found abundantly in the central nervous system (CNS; Wyse and Netto, 2011) where it contributes to the regulation of glutamate dependent transmission. Extracellular applications of L-proline at physiological concentrations enhance glutamatergic transmission at hippocampal synapses (Cohen and Nadler, 1997) and elevated concentrations of L-proline depolarize hippocampal neurons by directly activating N-methyl-D-aspartate (NMDA) and α-amino-3-hydroxy-5-methyl-4-isoxazolepropionic acid (AMPA) receptors (Martin et al., 1992; Pace et al., 1992). Animals suffering from metabolic disorders caused by defective proline dehydrogenase 1 (PRODH), the mitochondrial enzyme that initiates the proline catabolic pathway, show increased concentration of L-proline in brain that leads to enhanced synaptic activity in hippocampus (Paterlini et al., 2005) and to behavioral phenotypes associated with mental illness, such as altered performance in cognitive tasks, sensorimotor gating and locomotor activity (Kanwar et al., 1975; Hayward et al., 1993; Paterlini et al., 2005). In humans, abnormally elevated levels of L-proline in hyperprolinemia patients have been reported to cause epilepsy, seizures and impaired cognitive function (Roussos et al., 2009), as well as to be associated with schizoaffective disorders (Jacquet et al., 2005) and schizophrenia (Clelland et al., 2011; Orešič et al., 2011).

Although no evidence exists for specific L-proline receptors in the CNS, L-proline fulfills several of the classic criteria used to define well characterized amino acid neurotransmitters, such as biosynthesis in synaptosomes, release after K^+^ induced depolarization and specific regional distribution in the brain (Malandro and Kilberg, 1996). The role of L-proline as neuromodulator in excitatory synaptic transmission is strengthened by the presence of the brain-specific, high-affinity L-proline transporter PROT (*Slc6A7*). PROT shows significant sequence homology and shared structural features with the large family of Na^+^/Cl^−^ dependent neurotransmitter transporters, which includes transporters for glycine, GABA, norepinephrine, dopamine and serotonin (Fremeau et al., 1992). Pharmacological and genetic studies have demonstrated the importance of these transporters modulating neurotransmission by translocating neurotransmitters from the extracellular environment into the cytoplasm of the cells expressing those carriers. For most transmitter systems, like glutamate, glycine and monoamines, the high affinity uptake is essential to reduce the neurotransmitter concentration in the extracellular space and synaptic clefts. Moreover, it supports the presynaptic intracellular accumulation of neurotransmitter that is crucial for efficient transmitter loading of synaptic vesicles (Kristensen et al., 2011). Immunohistochemical studies revealed that PROT is localized presynaptically in subpopulations of glutamatergic neurons, suggesting that PROT functionally regulates the reuptake of L-proline and possibly also the levels of released L-proline at specific glutamatergic nerve terminals (Crump et al., 1999; Renick et al., 1999). In these synapses, PROT is thought to play a vital role both controlling the ability of L-proline to potentiate excitatory transmission in the vicinity of the transporter and limiting the extracellular concentration of L-proline to ensure that it does not reach levels that would inappropriately activate glutamate receptors (Renick et al., 1999). Supporting this hypothesis, PROT mutations and *Slc6A7* expression changes have been related to susceptibility to autism as well as schizophrenia (Hager et al., 2005; Voineagu et al., 2011; Hedges et al., 2012; Wen et al., 2014), and microdeletions at the 5q32 locus containing the *Slc6a7* gene have been associated with intellectual disability (Vincent et al., 2014). However, despite substantial knowledge of PROT function gained in cell culture systems, its physiological relevance remains unknown. Here we generated and characterized a PROT knockout (KO) mouse line to gain insights into its *in vivo* function. PROT deficient mice exhibited endophenotypes associated with mental illness, such as reduced locomotor activity, decreased approach motivation and impaired memory extinction, as well as alterations of glutamatergic synapse biochemistry in cortex and thalamus. Our study identifies a role for PROT in regulating behaviors that are needed to respond to environmental changes.

## Materials and Methods

### Ethics Statement

All animal experiments were performed according to the German law and were approved by the government offices in Ansbach, Würzburg and Recklinghausen, Germany. Unless stated otherwise, mice were kept at a constant night/day cycle (12 h/12 h) with access to water and food *ad libitum*. All efforts were made to minimize the suffering and number of animals used.

### Generation of PROT^−/−^ Mice

**Figure 1:**
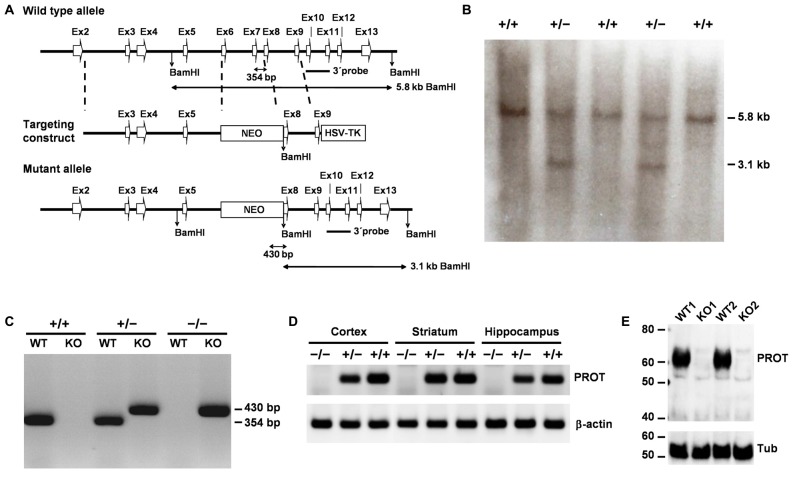
Targeted disruption of the mouse PROT gene. **(A)** Knockout (KO) strategy showing the wild-type (WT) PROT locus, the targeting construct, and the targeted allele. Exons are represented as white arrow boxes. The probe used for Southern analysis, the sizes of the restriction fragments detected with this probe, and the sizes of the bands generated by PCR analysis of genomic tail DNA are indicated. **(B)** Southern blot analysis of DNA isolated from untargeted (+/+) and targeted (+/−) embryonic stem (ES) cell clones. As expected, the BamHI restriction fragment size change from 5.8 kb to 3.1 kb is seen in the targeted cells. **(C)** Genotyping of F2 mouse offspring using PCR analysis of genomic tail DNA. The 354- and 430-bp bands represent the WT and mutant PROT alleles, respectively. **(D)** Reverse transcriptase-polymerase chain reaction (RT-PCR) analysis of brain region expression of PROT and β-actin in WT (+/+), heterozygous (+/−), and homozygous (−/−) KO mice. Note the absence of PROT transcripts in homozygous animals. **(E)** Western blot analysis of cortex membrane fractions from different animals using the PROT antibody and, after stripping, reprobed with the β-tubulin antibody. Note that PROT protein expression is abolished in the homozygous mutant mice.

A C57BL/6J mouse bacterial artificial chromosome (BAC) library was virtually screened with the sequence of the PROT gene (*Slc6a7*) using the NCBI clone finder. Three clones containing PROT genomic DNA were obtained from the Children’s Hospital Oakland Research Institute (CHORI), seeded on LB plates and grown to recover BAC DNAs. Southern blot analysis with a PCR fragment corresponding to exons 3 and 4 of the gene as a probe confirmed the presence of all coding exons of the *Slc6a7* gene in the three clones. Based on these clones, the targeting vector was constructed from a 7.3 kb genomic sequence, including exons 2–9, in which a 1.15 kb fragment encompassing exons 6 and 7 was replaced with the 1.8 kb PGK-neomycin resistance (*NEO*) gene. A 2.8 kb herpes simplex virus thymidine kinase gene fragment was attached to the 3′ end of the construct for negative selection (Figure 1A). The targeting vector was linearized at a unique NotI site and introduced into HM1-mouse embryonic stem (ES) cells by electroporation, followed by selection in G418 and FIAU. Colonies surviving the double selection procedure were isolated and screened by Southern blotting for homologous recombination using a 3′ external probe. Five positive ES cell clones were injected into C57BL/6 blastocysts and two generated chimeras were then backcrossed with female C57BL/6 mice. Genomic DNA prepared from tail biopsies of offspring was analyzed by PCR to identify correct insertion of the neo gene. The wild-type (WT) allele was detected using primer WTSIII (5′-AGAACATCTACAGGTCAGTG-3′) and primer WTAS (5′-GCTATCCCCCATCTTGCCTG-3′) which amplifies a fragment of 354 bp. Presence of the KO allele was detected using the primer neoS, located in the *NEO* gene (5′-CAGCTCATTCCTCCCACTCATGAT-3′), and primer WTAS, yielding a 430 bp fragment. The identified heterozygous offspring were crossed to yield homozygous mutant animals.

### Reverse Transcriptase-Polymerase Chain Reaction (RT-PCR)

Mice were sacrificed by cervical dislocation and brains were removed rapidly. Total RNA samples were isolated from the indicated brain tissues using the RNeasy^®^ Micro Kit (Qiagen) according to the manufacturer’s instructions. The RNA samples were treated with DNase I (Thermo Fisher Scientific), and cDNA was synthesized with the iScript™ cDNA synthesis Kit (BioRad). Reverse transcriptase-polymerase chain reaction (RT-PCR) reactions were done on a T3 Thermocycler (BioRad) using the GoTaq^®^ Flexi DNA Polymerase (Promega) according to the supplier’s instructions. The following sense (s) and antisense (as) primers were used: PROT, OL-5 (s) 5′-GTTCCTCTGTATCTTGAAGG-3′ (exon5) and OL-8 (as) 5′-CTGAGACATGTAGCCCAGCA-3′ (exon8), both oligos flanking the selection *NEO* marker cassette and yielding a 355 bp fragment; β-actin, OL-ActinS (s) 5′-TGTTACCAACTGGGACGACA-3′ and OL-ActinAS (as) 5′-GGGGTGTTGAAGGTCTCAAA-3′, yielding a 167 bp PCR band.

### Brain Membrane Preparation

Mice of both genders (approximately 12 weeks old) were sacrificed by cervical dislocation and brains were removed rapidly and dissected. The tissue samples were homogenized in 1 ml of ice-cold isolation medium (0.33 M sucrose, 1 mM EDTA, 1 mM phenylmethylsulfonyl fluoride, 10 mM HEPES-Tris [pH 7.4]) using a Dounce-type glass homogenizer. The homogenate was centrifuged (1,000× *g*, 5 min) at 4°C, the pellet discarded, and the supernatant centrifuged at 17,000× *g* for 10 min. The resulting crude synaptosomal fraction pellet (P2) was washed once and resuspended in modified Krebs-Henseleit medium (125 mM NaCl, 5 mM KCl, 2.7 mM CaCl_2_, 1.3 mM MgSO_4_, 10 mM glucose, 25 mM HEPES-Tris [pH 7.4]). Protein concentration was determined using the Pierce BCA Protein Assay (Thermo Scientific) according to manufacturer’s instructions.

### Neuronal Cell Culture and Lysate Preparation

Primary neuronal cultures were prepared from neonatal (postnatal day 0–1) mouse cortex. Animals were sacrificed by decapitation and the brains were dissected in ice cold phosphate buffered saline solution (PBS). Cortical tissues were digested with papain (1.0 mg/ml in PBS [Ca^2+^ and Mg^2+^ free] with 0.2% glucose and 0.1% BSA) at 37°C for 30 min, dissociated in trituration buffer (HBSS [Ca^2+^ and Mg^2+^ free] with 0.1% glucose and 10 mM HEPES [pH 7.4]), and transferred to poly-L-lysine (0.1 mg/ml; Sigma) coated dishes in plating medium (MEM containing 10% fetal calf serum, 0.45% glucose, and 100 Uml^−1^ penicillin/streptomycin; Invitrogen), plated at a density of 0.1 × 10^6^ cells/cm^2^. After 5 h, medium was changed to maintenance medium (Neurobasal medium supplemented with 2 mM glutamine, 2% B-27 and 100 Uml^−1^ penicillin/streptomycin; Invitrogen). Cytosine arabinoside at a final concentration of 5 μM was added 2 days after plating to the medium to curtail glia growth in the culture. Neuronal cultures were fed twice per week by aspirating half the medium from each well and replace it with fresh maintenance medium warmed to 37°C until use ~2 weeks old.

For lysate preparations, cells cultured in 6-well plates were washed twice with ice-cold PBS and lysed in ice-cold lysis buffer (150 mM NaCl, 1 mM EDTA, 1% Triton X-100, 1% IGEPAL, 25 mM Tris, pH 7.4) supplemented with protease inhibitor mixture (Sigma). Lysates were rotated 30 min at 4°C and centrifuged at 15,000× *g* at 4°C for 10 min. Protein concentration was determined using the Pierce BCA Protein Assay (Thermo Scientific) according to manufacturer’s instructions.

### Transport Assays

20 μl aliquots of the brain membrane suspension (equivalent to 30–50 μg of protein) were preincubated for 2 min at 37°C. Uptake was initiated by addition of 80 μl of a modified Krebs-Henseleit solution kept at 37°C containing either [3,4-^3^H]L-glutamic acid (0.1 μM) or [2,3,4,5-^3^H]L-proline (2 μM, both Hartman Analytic). After a 1 or 2 min incubation, respectively, with gentle agitation, uptake was terminated by diluting the incubation mixture with 3 ml of modified Krebs-Henseleit medium at 4°C followed by rapid filtration through a moistened filter (SM 11106, 0.45 μm pore size; Sartorius). Filters were rinsed twice with 3 ml of modified ice-cold Krebs-Henseleit medium. All dilution, filtration and washing procedures were performed within 15 s. Filters were dried and placed in microvials, and their radioactivity measured by scintillation spectrometry.

Uptake in neurons was performed after the cells cultured in 24-well plates were maintained for 14 days in culture. Medium was removed by aspiration and, after washing with PBS (with Ca^2+^ and Mg^2+^) at 37°C, cells were maintained for 15 min in 200 μl of PBS containing 6 mM-glucose in the presence or absence of 50 μM des-tyrosyl-leu-enkephalin (GGFL; Phoenix Pharmaceuticals). Uptake was started by adding radioactive L-proline (2 μM final concentration). The Na^+^ dependance of L-proline uptake was determined by isotonic substitution of assay NaCl with LiCl. Cells were incubated for the indicated times, and then uptake was terminated by washing the cells with 3 × 500 μl of ice-cold PBS. Cells were solubilized with 250 μl of 0.2 M NaOH. Samples (200 μl) were placed directly in microvials to measure their radioactivity by scintillation spectrometry, and protein concentration (30 μl) was determined.

### Western Blotting

Samples (10–20 μg of protein) were separated by 10% SDS-polyacrylamide gel electrophoresis and transferred to nitrocellulose membrane (HybondTM-C Extra, GE Healthcare) by electroblotting. After washing, membranes were blocked with Roti-Block (1×; Carl Roth) for 1 h at room temperature and incubated overnight at 4°C in primary antibody solution in Roti-Block. Antibodies to detect calcium/calmodulin-dependent protein kinase II alpha (CaMKIIα), GluA1, GluA3, phospho-CaMKII, GluK5 and PSD95 were from Merck Millipore. The GluN2A and GluN2B antibodies were from Cell signaling Technology, the GluN1A and Catechol-O-Methyltransferase (COMT) antibodies were from BD Bioscience, the GluK3 antibody was from ABIN, and the GluA2 antibody was from Biozol. PROT-specific rabbit polyclonal antiserum was raised against residues 597–620 from the predicted COOH terminus of mouse PROT (GSQSPKPLMVHMRKYGGITSFENT). Membranes were washed three times with PBS containing 0.1% Tween and then incubated for 1 h at room temperature with a horseradish peroxidase-conjugated secondary antibody goat anti-rabbit IgG Antibody HRP (ABIN) or goat anti-mouse IgG antibody HRP (Sigma) in Roti-Block. The immunoreactive proteins were visualized by chemiluminescence using Amersham Biosciences ECL Prime Western blotting detection reagent (GE Healthcare) and quantified by densitometry using Gelscan software (Bioscitec, Frankfurt, Germany). To normalize for equal loading and protein transfer, membranes were reprobed with an antibody against β-tubulin (ABIN).

### Histological Analysis

Mice (approximately 12 weeks old, 3 animals per genotype) were anesthetized, sacrificed and brain cryostat sections were prepared and fixed with 4% (w/v) paraformaldehyde in standard PBS (pH 7.4) and stained with 0.1% (w/v) cresyl violet, rinsed twice in water to remove excess stain, desiccated by washing with increasing concentrations of ethanol, cleared in xylene and embedded in xylene based embedding medium.

### Behavioral Tests

Behavioral studies were carried out with WT and KO male mice in the present study. The animals were approximately 12 weeks old at the beginning of the behavioral testing.

#### Eight-Arm Radial Maze

Animals were singly housed and gradually reduced to 85% of their normal body weight by being fed only limited amounts of chow daily. The mice were maintained at reduced weight throughout the experiment. The apparatus was an opaque plastic maze with eight identical arms radiating from an octagonal starting platform. The mice were pre-trained for 3 days (one 15 min trial per day) by placing the mice in the central starting platform and allowing them to explore and to consume food pellets located at the distal end of each arm. The maze trials started at day 4. All eight arms contained a food pellet at the beginning of each trial and the baits were not replaced once they had been taken. Animals received one trial per day (19 trials in total); each daily trial terminated when eight correct choices were made or 15 min had elapsed. A correct choice was recorded when the mouse entered an unvisited arm and took the food pellet during the trial, while re-entering into an already visited arm from which the food had already been taken was counted as an error.

#### Acoustic Startle Response and Pre-pulse Inhibition

Acoustic startle responses and pre-pulse inhibition (PPI) of startle responses were assessed using a standard startle chamber (TSE-Systems, Bad Homburg, Germany). Mice were placed in the startle chamber and left undisturbed for 5 min prior to testing in the presence of a 60 dB background noise. Testing consisted of 60 random 110 dB pulses that included 10 pulses alone and 50 pulses preceded by prepulses of 65-, 70-, 75-, 80- or 85-dB. The prepulse sounds were presented 50 ms before the startle stimulus. Maximal startle amplitude in response to the startle stimulus was determined. The average intertrial interval was 15 s (from 10 s to 20 s). PPI was calculated as a percentage score: PPI (%) = (1 −[(startle response for pulse with prepulse)/(startle response for pulse alone)]) × 100.

#### Open Field

Locomotor activity in the open field was examined using a brightly lit arena (50 cm × 50 cm × 50 cm, 200 lux). Mice were placed in the periphery of the arena and then their locomotor activity during 30 min was recorded and analyzed using the VIDEOMOT video tracking system (TSE Systems). The number of visits to the central area (infield square of 26 × 26 cm) as well as the path length speed and resting time were recorded.

#### Rotarod

Motor coordination and balance were tested on an accelerating rod apparatus (TSE Systems). Mice were placed on a rotating rod (3 cm diameter) and the time each animal was able to maintain its balance on the rod was determined. The speed of the rotarod accelerated from 2 rpm to 20 rpm over a 5 min period. Mice were pre-trained for one trial on the accelerating rod prior to the test.

#### Tail-Suspension Test

Mice were suspended individually by their tail from a metal rod. The rod was fixed 50 cm above the surface of a table covered with soft cloth in a sound-isolated room. The tip of the tail was fixed using adhesive Scotch tape; the duration of the test was 6 min. The immobility time was determined by an observer, using a stopwatch, who was unaware of the genotype.

#### Forced-Swimming Test

Mice were placed in a Plexiglas cylinder (10 cm internal diameter, 50 cm high) filled with 24°C water (10 cm height). Duration of the experiment was 6 min and the behavior of the animals was evaluated between the first and sixth minute for 5 min. The immobility time was measured by an observer, who was unaware of the genotype, using a stopwatch. A mouse was judged to be immobile when it remained floating in the water, making only those movements necessary to keep its head above the water.

#### Elevated Zero-Maze

Mice were kept in the normally illuminated room where experiment was performed and 1 h later their activity on the zero-maze was measured for 5 min. The maze consisted of an annular white platform (inner diameter 46 cm, 5.6 cm width) elevated 40 cm above ground level and divided equally into four quadrants. The two opposite quadrants were enclosed by white walls (24 cm high) on both edges of the platform. The behavior of mice was videotaped and analyzed with the VIDEOMOT video-tracking system (TSE Systems). Distance traveled in the open and closed parts, as well as numbers of visits and time spent in the open area were evaluated.

#### Response to a Novel Object

After a 15 min period of habituation to an empty open field arena, a novel object (a 355 mL beverage can) was placed in the center. Total distance traveled as well as number of approaches and time spent in the proximity (26 × 26 cm central region) of the object were monitored using the VIDEOMOT video tracking system.

#### Morris Water Maze

Animals were habituated in the room for 30 min prior to the experiment. For pretraining, mice were put in a circular pool (160 cm in diameter) filled with clean water (24°C) to find a platform (10 × 15 cm) that was visible 1 cm above the water. They were allowed to sit on the platform for 30 s after climbing onto it or being manually guided to the platform. During the training period the platform was hidden 1 cm below water surface. The water was dyed with nontoxic white color so that the platform was no longer visible. For each trial, a random position was selected as a starting point. The mouse was gently put into the water and the time as well as the distance required to find the platform was measured using the software VIDEOMOT. The maximal testing period was 1 min. In case the mouse did not find the platform within this period, it was put gently on the platform for 20 s and then back to its home cage. Mice that found the platform on its own were put immediately back into their home cage. Each mouse was tested twice a day during nine consecutive days. A probe test was performed 24 h after the last day of training. Forty-eight and 72 h after the probe test, two extinction trials were performed. The platform was located into the opposite quadrant, and the behavior of the mice was recorded and analyzed with VIDEOMOT video tracking system.

### Statistical Analysis

Data are presented as means ± SEM or SD, and calculations were performed using GraphPad Prism 6 (GraphPad Software). Comparisons were analyzed with a two-tailed Student’s *t* test between two experimental groups, and with one-way analysis of variance (ANOVA) with Bonferroni’s *post hoc* test between more than two groups. Comparisons of one measurement variable between WT and PROT KO groups were carried out using two-way ANOVA with Bonferroni’s post-test. *P* value significance thresholds were *P < 0.05, **P < 0.01 and ***P < 0.001.

## Results

### Generation of PROT Knockout Mice

We inactivated the PROT gene (Yates et al., 2016) in mouse HM1 (129/OLA) ES cells via homologous recombination. The central region of PROT, which included the transmembrane domain 5, the extracellular loop 3 and the transmembrane domain 6, was replaced by a neomycin-resistance cassette (Figure 1A). Targeted ES cell clones were identified by Southern blot analysis, exploiting a newly introduced BamHI site (Figure 1B). This analysis showed that 10 out of a total of 106 screened clones had been targeted properly. Two male chimeras obtained from properly targeted ES cell clones were then crossed with C57/BL6 female mice to establish germline transmission of the mutation. Mice heterozygous (PROT^+/−^) appeared phenotypically normal and showed undisturbed development and fertility. Intercrossing of the heterozygous mice generated PROT deficient (PROT^−/−^) animals (Figure 1C) at normal Mendelian ratios, indicating no increased embryonic mortality in the homozygous mice. RT-PCR experiments showed absence of PROT mRNA in forebrain regions of PROT^−/−^ animals (Figure 1D) and Western blot analysis failed to reveal immunoreactive PROT protein in the homozygous mice (Figure 1E), thus confirming lack of PROT expression in PROT KO mice. To minimize the influence of genetic background in our analyses, we subsequently introduced the PROT deletion into the C57BL/6 strain through backcrossing for at least eight generations. PROT^−/−^ mice showed undisturbed development and fertility as well as normal appearance of fur and whiskers.

### Reduction of Synaptosomal Accumulation of L-Proline Does Not Lead to Neuronal Toxicity in PROT^−/−^ Mice

**Figure 2:**
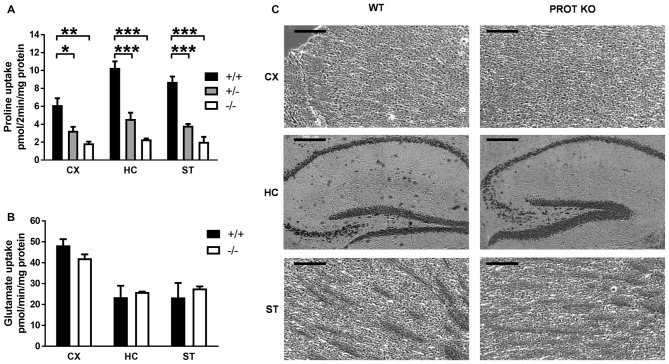
L-Proline transport activity is reduced in PROT^−/−^ mice. **(A,B)** Membranes were prepared from the indicated tissues of WT (+/+), heterozygous (+/−), and homozygous (−/−) KO mice, and then assayed for L-proline uptake for 2 min **(A)** or L-glutamate uptake for 1 min **(B)** at 37°C. **(C)** Cresyl violet-stained sections from WT and PROT KO mice are shown. No defects were detected in the homozygous mutants, and the overall anatomy appeared normal in all animals analyzed. CX, cortex; HC, hippocampus; ST, striatum. Data are shown as means ± SEM (*n* = 3 for each genotype). **(A)** One-way analysis of variance (ANOVA) with Bonferroni’s *post hoc* test; **(B)** two-tailed Student’s *t* test; *p < 0.05; **p < 0.01; ***p < 0.001. Scale bars: 200 μm.

To estimate the quantitative contribution of PROT to synaptosomal proline accumulation, high-affinity proline uptake was measured in crude membrane fractions prepared from forebrain regions known to express PROT. As shown in Figure 2A, L-proline uptake was significantly reduced by ≥75% in the cortex, hippocampus and striatum of PROT^−/−^ mice as compared to WT littermates. Samples from heterozygous animals showed intermediate uptake values, consistent with a gene dosage effect of PROT expression. Conversely, all genotypes showed comparable L-glutamate uptake values, indicating no gross alterations in the expression of glutamate transporter at excitatory presynaptic terminals (Figure 2B). These results are consistent with a complete lack of PROT-mediated L-proline uptake in the homozygous mutant mice.

Larger concentrations of L-proline have been reported to be excitotoxic for pyramidal and granule cells in hippocampus, with neuronal toxicities comparable to those exerted by glutamate (Nadler et al., 1988). It might be conceivable that a similar neurotoxic effect could also arise in PROT deficient animals. Gross evaluation and histological examination showed that brain morphology of PROT^−/−^ mice was indistinguishable from that of WT littermates (Figure 2C), indicating that PROT deficiency does not result in a significant increase in cell death.

### PROT Knockout Mice Display a Phenotype Dissimilar From PRODH Mutant Animals

**Figure 3:**
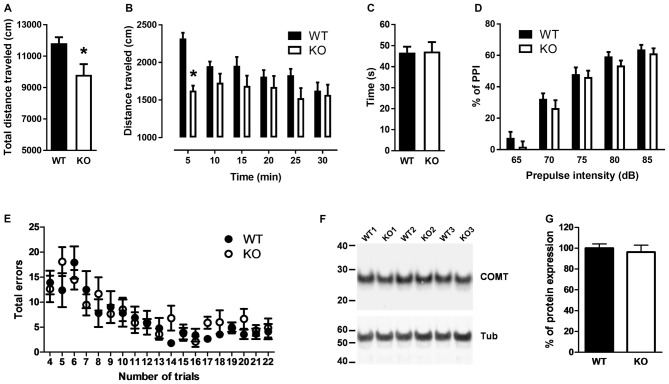
Behavioral analyses of WT and PROT^−/−^ (KO) mice. **(A,B)** Total distance traveled by WT and KO mice in the open field during a 30 min interval **(A)** and distance traveled over the 30 min period grouped in six 5-min intervals **(B)** revealed decreased locomotor activity in PROT-deficient mice (*n* = 13 mice for each genotype). **(C)** Rotarod test on WT and KO mice (*n* = 20 mice for each genotype) displayed no significant different performance at the rod. **(D)** Sensorimotor gating analysis in KO and WT mice assayed by the inhibition of an acoustic startle response (110 dB) with the indicated prepulse dB levels. Pre-pulse inhibition (PPI) (%) values are means ± SEM (WT, *n* = 24 mice; KO, *n* = 22 mice; *p* > 0.05, two-way repeated-measures ANOVA). **(E)** Performance of KO mice on the eight-arm radial maze displayed no memory deficits over the trial sessions compared to WT animals. Data represent the means ± SEM (WT, *n* = 10 mice; KO, *n* = 11 mice) of the number of revisiting errors in each trial. **(F,G)** Representative immunoblot examining Catechol-O-Methyltransferase (COMT) expression **(F)** and quantitative analysis of COMT immunoreactive band corrected by β-tubulin** (G)** of fractions prepared from the cortex of WT and KO mice (*n* = 11 mice for each genotype). Data are represented as means ± SEM. **(A)** Two-tailed Student’s *t* test; **(B,D)** two-way repeated-measures ANOVA with Bonferroni’s *post hoc* test; *p < 0.05.

Because elevated L-proline levels in brains of mice carrying a defective PRODH modify glutamatergic transmission that leads to deficits in learning and memory, reduced sensorimotor gating and impaired locomotor activity (Gogos et al., 1999; Paterlini et al., 2005), we reasoned that PROT deficient mice could display similar deficits. Consequently, we first monitored their baseline locomotor activity in the open-field test during 30 min. PROT deficient mice revealed depression of mobility in this paradigm (Figures 3A,B). This reduction was not due to impaired motor coordination and balance, since in the accelerating rotarod test PROT^−/−^ mice displayed comparable motor coordination skills to WT littermates (Figure 3C).

Sensorimotor gating was examined by the PPI test. In WT mice, a low-intensity pulse of sound preceding an acoustic startle stimulus inhibited their startle reflex. Although we observed a trend of the PROT^−/−^ mice to show lower PPI (Figure 3D), which would suggest some contribution of PROT for pre-attentive processing, no statistically significance was observed.

Cognitive ability was then analyzed by using the eight arm radial maze paradigm. PROT^−/−^ mice learned the maze task, in which the animals were required to collect eight rewards, during three consecutive days performing as well as their WT littermates. They also did not show any difference in the maze test during nineteen consecutive trials (Figure 3E), thus revealing no changes on learning acquisition and working memory performance in the mutant mice.

Taken together, our results show that, although PROT^−/−^ mice displayed a similar neuromotor depression as observed in PRODH mutant mice (Paterlini et al., 2005), there are major differences in the behavioral phenotype of both strains. Consistent with this interpretation, Western blot analysis of samples from PROT^−/−^ mouse cortices displayed no changes in COMT protein levels (Figures 3F,G), an effect observed in PRODH mutant mice as result of secondary dysregulation of dopaminergic neurotransmission (Paterlini et al., 2005).

### Synaptic Protein Expression in PROT Deficient Mice

**Figure 4:**
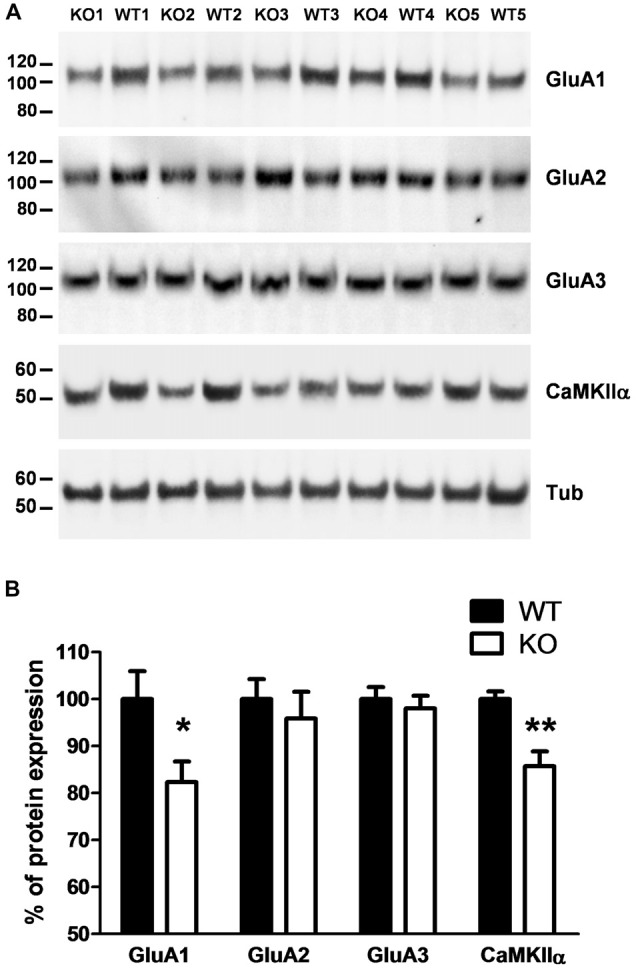
PROT^−/−^ mice show altered expression of synaptic proteins in cortex. **(A)** Western blot analysis of cortex crude synaptomes (P2) prepared from WT and PROT^−/−^ (KO) animals. Equal amounts of protein (20 μg/lane) were loaded and probed with the indicated antisera. **(B)** Quantitative analysis of the immunoreactive bands corrected by β-tubulin (*n* = 12 mice for each genotype; *p < 0.05; **p < 0.01; two-tailed Student’s *t* test). Data are shown as means ± SEM.

Because PROT KO mice only display a subset of the defects observed in PRODH deficient mice, we then investigated if homeostatic changes in the expression levels of proteins known to be involved in synaptic plasticity at glutamatergic synapses could be detected in PROT deficient mice that might affect their behavioral phenotype. Despite the restricted localization of PROT to a subset of glutamatergic neurons (Renick et al., 1999), Western blot analysis performed on crude synaptosomal fractions prepared from cortex revealed a decrease in the total expression level of the AMPA receptor subunit GluA1 in PROT deficient mice, whereas the expression levels of the AMPA receptor subunits GluA2 and GluA3 were unaltered (Figures 4A,B). Likewise, the expression level of CaMKIIα was diminished. Conversely, no major modifications on protein levels were identified in hippocampus (Table 1). In addition, we measured the expression levels of NMDA receptor subunits GluN1, GluN2A and GluN2B and the NMDA receptor anchoring protein PSD-95, as well as the kainate receptor subunits GluK3 and GluK5, and found no differences between WT and PROT KO littermates in both brain regions (Table 1). Our data suggest that in cortex of PROT deficient mice the decrease in GluA1 and CaMKIIα levels is a selective abnormality within those subpopulations of glutamatergic synapses where PROT is no longer expressed.

**Table 1:** Quantification of synaptic protein immunoreactivities.

	Cortex		Hippocampus	
	WT	KO	WT	KO
GluN1	100.0 ± 4.085	93.20 ± 6.924	100.0 ± 5.184	96.43 ± 6.361
GluN2A	100.0 ± 3.428	92.38 ± 5.551	100.0 ± 3.080	99.14 ± 6.056
GluN2B	100.0 ± 7.103	96.55 ± 7.746	100.0 ± 3.315	93.01 ± 3.654
PSD95	100.0 ± 3.342	92.88 ± 4.007	100.0 ± 5.386	102.3 ± 5.653
GluA1	100.0 ± 5.936	82.30 ± 4.404*	100.0 ± 3.262	100.1 ± 3.327
GluA2	100.0 ± 4.222	95.80 ± 5.706	100.0 ± 3.152	100.0 ± 4.462
GluA3	100.0 ± 2.573	98.01 ± 2.670	100.0 ± 3.852	99.47 ± 8.604
CaMKIIα	100.0 ± 1.618	85.71 ± 3.161**	100.0 ± 4.580	99.33 ± 3.535
GluK3	100.0 ± 6.193	95.45 ± 5.699	100.0 ± 8.115	102.0 ± 5.730
GluK5	100.0 ± 3.077	90.12 ± 4.143	100.0 ± 4.037	98.71 ± 3.088

Values were determined by scanning immunoreactive bands on Western blots and represent means ± SEM (n = 12 mice for each genotype). Significantly reduced band intensities found in samples from KO tissues are indicated by bold typesetting. *p < 0.05; **p < 0.01; two-tailed Student’s t test.

### PROT Influences GluA1 Receptor Levels

**Figure 5:**
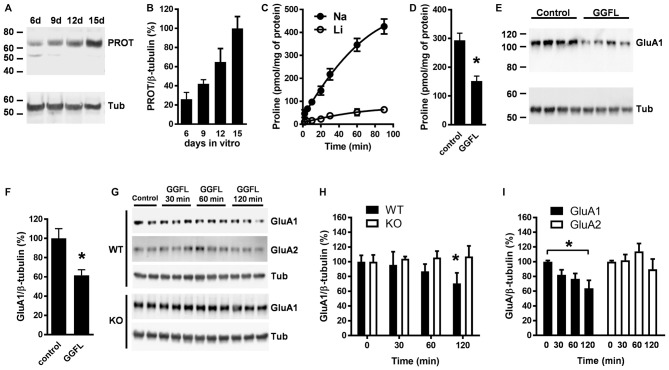
PROT influences GluA1 receptor levels in cultured cortical neurons. **(A)** Lysates prepared from mouse cortical neurons cultured for the period indicated (in days) were analyzed by Western blotting using the PROT antibody and reprobed with the β-tubulin antibody. **(B)** Quantitative analysis of PROT immunoreactive band corrected by β-tubulin from three independent experiments. **(C)** Time course of L-proline accumulation into cortical neurons cultured after 14 days; Na^+^ dependance was examined by isotonic substitution of assay NaCl (Na) with LiCl (Li). **(D)** Inhibition of L-proline transport by GGFL in cortical neurons; Na^+^ dependent L-proline uptake was conducted for 60 min in the absence or presence of 50 μM GGFL. **(E,F)** Treatment with 50 μM GGFL for 120 min diminished GluA1 expression in lysates from cortical neurons. **(G)** Illustrative time-dependent Western blot assay of GluA1 and GluA2 abundance from cortical neuron lysates prepared from WT as well as of GluA1 from PROT^−/−^ (KO) mouse, with or without 50 μM GGFL. **(H)** Summary of GluA1 quantification obtained in mouse neuron lysates from three different experiments. **(I)** GluA2 expression is not affected in WT neurons upon incubation with GGFL. Data are presented as means ± SD. **(D,F)** Two-tailed Student’s *t* test; **(H)** two-way ANOVA with Bonferroni’s *post hoc* test; **(I)** one-way ANOVA with Bonferroni’s *post hoc* test; *p < 0.05.

To examine whether PROT inhibition affects GluA1 receptor expression, we prepared primary cortical neurons from early postnatal WT mouse cortex. Western blotting analysis detected PROT after 6 days of culture, increasing its expression during the following days in culture (Figures 5A,B). Consequently, two-week-old cultured neurons accumulated L-proline in a time-dependent manner (Figure 5C). Treatment with des-tyrosyl-leu-enkephalin (GGFL), an opiate receptor-inactive peptide previously described as selective inhibitor of PROT (Galli et al., 1999), decreased L-proline uptake (Figure 5D). Immunoblotting analysis of lysates prepared from neurons incubated with GGFL showed that the expression level of the GluA1 subunit of the AMPA receptor was reduced compared with untreated controls (Figures 5E,F). Specificity of the modulation of AMPA receptor expression by PROT was confirmed by time course analyses of GGFL treatment, displaying a gradual decrease of GluA1 levels in samples from WT neurons whereas no alteration was observed when GGFL was incubated in cortical neurons prepared from PROT deficient mice (Figures 5G,H). Furthermore, no significant changes in the expression of GluA2 were detected in WT neurons upon GGFL incubation (Figure 5I).

### PROT Modulates Emotional Behavior Responses Related to Novelty Seeking

**Figure 6:**
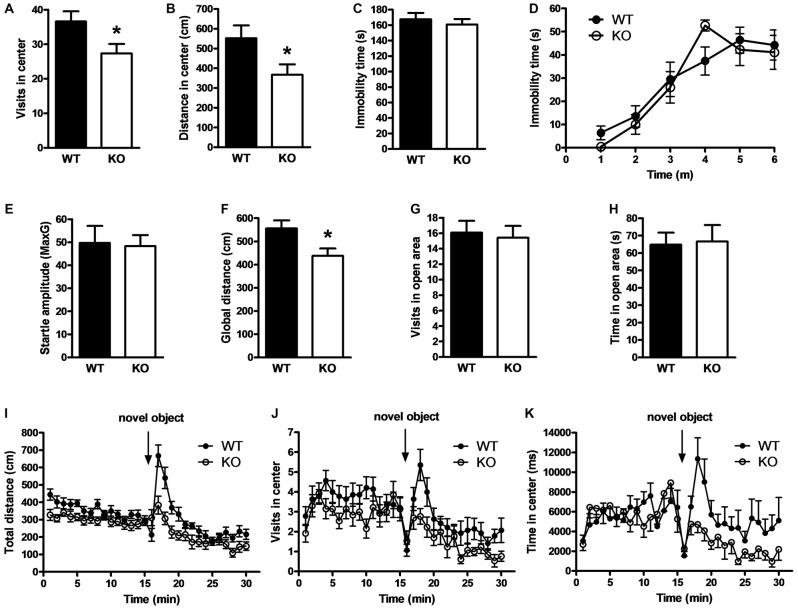
Effects of PROT deletion on emotional behavior in PROT^−/−^ mice. **(A)** Locomotor activity analyses in the central part of the open-field for 15 min displayed reduced number of entries for PROT^−/−^ (KO) mice (*n* = 52) compared with WT animals (*n* = 56). **(B)** Similar analyses revealed diminished distance traveled in the center for the KO (*n* = 39) in contrast to WT (*n* = 42). **(C,D)** Behavior of WT and KO mice in behavioral despair models of depression; both in the tail suspension test **(C)** and the forced-swimming test **(D)** WT (*n* = 10) and KO (*n* = 7) animals spent similar time immobile. **(E)** Startle response test for characterization of anxiety-related behavior; the basal startle amplitude in KO mice was comparable to that of WT (*n* = 10 mice for each genotype). **(F–H)** Zero-maze test; KO mice (*n* = 17) showed diminished locomotor activity compared with WT (*n* = 14) animals **(F)** during the 5 min trial, but no alterations in the number of entries **(G)** and time spent **(H)** into the open sector. **(I–K)** PROT KO mice display reduced locomotor responses to a novel object; KO (*n* = 13) exhibited decreased locomotor activity in response to a novel object **(I)**, performed less entries **(J)**, and spent less time **(K)** in the proximity of the novel object compared with WT (*n* = 14). Data are depicted as means ± SEM. *p < 0.05; two-tailed Student’s *t* test.

Because PROT deficiency led to subtle changes in glutamatergic synaptic protein expression, we wondered whether behavioral differences might be observed under more demanding challenges. Therefore, we carried out new open field tests to evaluate mouse emotional behavior. We observed that PROT^−/−^ mice entered (Figure 6A) and moved less (Figure 6B) in the central area of the arena compared with WT littermates, thus displaying a lower exploratory behavior.

Animal models of depression show similar reduced exploratory behavior (Bilkei-Gorzo et al., 2002). However, quantification of mouse activity in the tail-suspension test displayed no alteration of immobility time in PROT deficient animals compared to WT littermates (Figure 6C). Likewise, no differences in immobility time were detected in the forced-swimming test (Figure 6D), thus displaying no depression-like behavior.

The open field arena is theorized to create a conflict by concurrently evoking both avoidance behavior, related to animal’s anxiety due to novelty, and approach behavior, which reflects animal’s tendency to explore novel stimuli or environments (Dulawa et al., 1999). Accordingly, mice were analyzed in the startle-reactivity test for increased anxiety (Bilkei-Gorzo et al., 2004). However, no change was observed in the amplitude of the startle responses (Figure 6E). Additionally, analysis of PROT deficient animals in the elevated zero maze (Shepherd et al., 1994) showed a reduction in total locomotion (Figure 6F), confirming the lower motor performance detected in the open field paradigm, but no variations in number of entries (Figure 6G) and time spent in the open areas of the annular platform (Figure 6H). Taken together, our results did not support anxiety-like behavior in the homozygous animals.

To test the exploratory drive of PROT^−/−^ mice, a free exploration paradigm was next carried out, the novel object test (Wiedholz et al., 2008). Mice were first habituated to the open field arena and then a novel object was placed in the center. WT mice showed characteristic exploratory behaviors such as approach, sniffing and direct contact to the novel object. However, PROT^−/−^ mice were less responsive as they displayed lower increase in total distance (Figure 6I) as well as reduced number of approaches to the novel object (Figure 6J) and less time spent in the central quadrant (Figure 6K) to explore the novel object. Hence, depressed approach behavior appears to account for the phenotype previously observed in the central area of the open-field paradigm. Our data suggest that PROT is involved in modulating emotional behavior responses related to novelty-seeking in mice.

### PROT Deletion Induces Synaptic Changes at Glutamatergic Synapses in Thalamus

**Figure 7:**
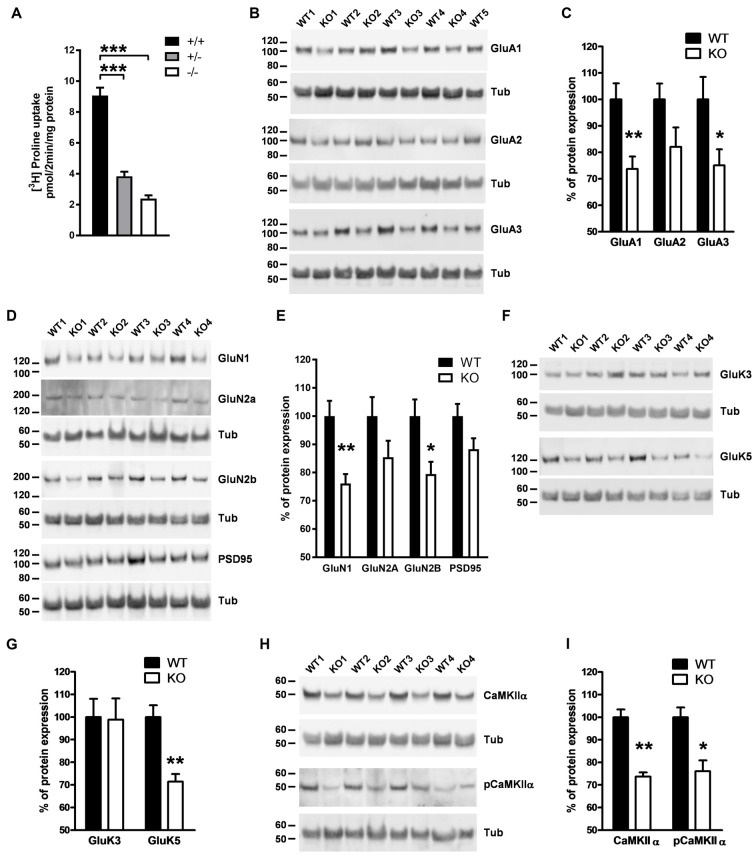
Analysis of components of glutamatergic synapses in thalamus of PROT deficient mice. **(A)** L-proline uptake is reduced in PROT^−/−^ thalamus; membranes were prepared from thalamus of WT (+/+), heterozygous (+/−), and homozygous (−/−) KO mice, and assayed for L-proline transport activity for 2 min. **(B–I)** Crude synaptosomes (P2) prepared from the thalamus of WT and PROT deficient (KO) animals (*n* = 11 mice for each genotype) were analyzed by Western blotting; illustrative Western blots and quantitative analyses of immunoreactive band abundance corrected by β-tubulin for α-amino-3-hydroxy-5-methyl-4-isoxazolepropionic acid (AMPA) receptor subunits **(B,C)**, N-methyl-D-aspartate (NMDA) receptor subunits and PSD95 **(D,E)**, kainate receptor subunits **(F,G)** and calcium/calmodulin-dependent protein kinase II alpha (CaMKIIα) **(H,I)** are depicted. Data are presented as means ± SEM. **(A)** One-way ANOVA with Bonferroni’s *post hoc* test; **(C,E,G,I)** two-tailed Student’s *t* test; *p < 0.05; **p < 0.01; ***p < 0.001.

The thalamus plays a vital role in a wide range of cognitive functions. Lesion or stimulation of this brain region leads to changes in emotional reactivity (Kirouac, 2015; Ouhaz et al., 2015). Since we observed alterations in emotional behavior in PROT^−/−^ mice, we then analyzed whether modifications in glutamatergic synapse biochemistry could be also detected in the thalamus as the previously observed in cortex. High-affinity L-proline uptake was significantly reduced by >70% in crude synaptosomal fractions prepared from thalamus of PROT^−/−^ mice as compared to WT littermates (Figure 7A), confirming the involvement of PROT on L-proline synaptic accumulation in this brain region. Western blot analyses of crude synaptosomal preparations revealed a decreased expression of the GluA1 subunit, but not of GluA2, of the AMPA receptor in PROT deficient mice. Additionally, a lower expression of the GluA3 subunit was also observed (Figures 7B,C). Moreover, the expression levels of the GluN1 and GluN2B subunits of the NMDA receptor were diminished, whereas no significant statistical variations were found on the expression levels of the GluN2A subunit and PSD95 (Figures 7D,E). We also detected a reduced expression of the high-affinity kainate receptor subunit GluK5, but not of the low-affinity GluK3 subunit (Figures 7F,G). Furthermore, we found that levels of CaMKIIα were diminished, suggesting a lower total activity for this kinase. Accordingly, lower levels of phosphorylated CaMKIIα were also detected (Figures 7H,I). Altogether, these results point to a significant role of PROT in the modulation of specific components of the glutamatergic synapses in thalamus.

### PROT Knockout Mice Exhibit Impairment in Memory Extinction

**Figure 8:**
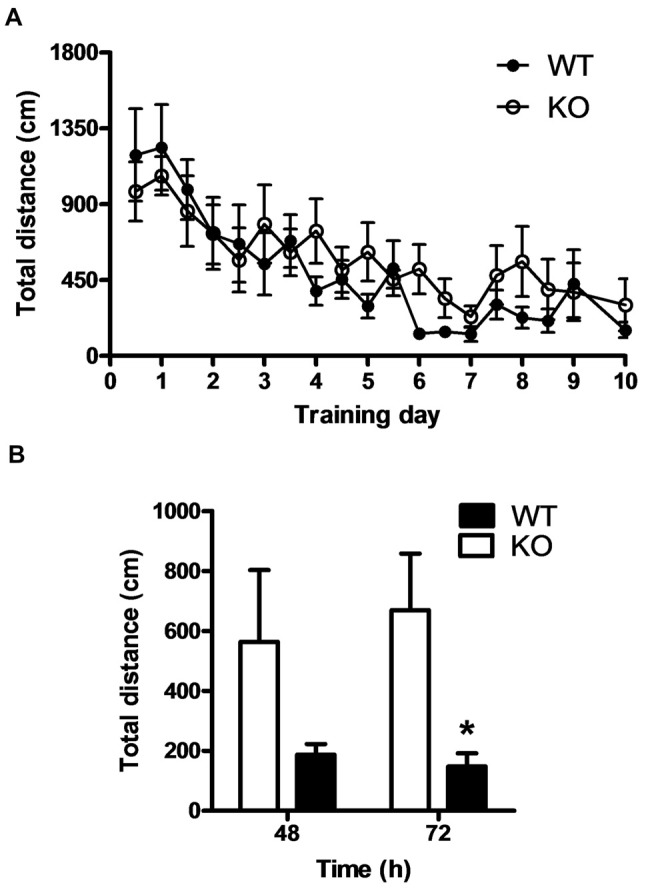
PROT^−/−^ mice exhibit normal memory acquisition but impaired memory extinction. **(A)** Compared to WT, PROT deficient mice (KO) showed normal spatial reference memory acquisition in the Morris water maze during the training period and the probe trial at day 10. **(B)** KO mice displayed impaired memory extinction; after the hidden platform was moved to the opposite quadrant of the maze, KO animals persevered searching for the platform in the former target quadrant and did not show decrease in their path length as compared with WT. Data are shown as means ± SEM; *n* = 6 for each genotype; **(B)** two-tailed Student’s *t* test; *p < 0.05.

Behaviors that allow responding to a constantly changing environment, such as novelty seeking, are essential for animal surviving. A comparable behavior is cognitive flexibility, which is the ability to shift from a learned to a different response when task contingencies change. We wondered whether the absence of PROT might also alter mouse mental flexibility. Therefore, a reversal learning test in the Morris water maze was performed. PROT KO and WT littermates were subjected to two training trials per day for 9 days and the path length was scored for each trial. A probe trial was conducted 24 h after the last day of training. We observed no significant differences between the groups during either the training or the probe trials (Figure 8A), confirming again that memory acquisition was unaffected by loss of PROT. Then, the hidden platform was moved to the opposite quadrant of the maze. WT mice actively unlearnt the preceding memory (extinction) to find the platform in the novel position, as evidenced by their decreased path length while searching for and localizing the new location of the platform 48 and 72 h after the probe trial. However, PROT deficient animals persevered searching for the platform in the former target quadrant, as demonstrated by their increased path length compared with the WT littermates (Figure 8B). We concluded that PROT^−/−^ mice exhibited deficits in memory extinction, without affecting initial learning acquisition.

## Discussion

The amino acid L-proline is known to modulate neurotransmission at glutamatergic synapses. Physiological levels of L-proline have been shown to potentiate excitatory transmission in rat hippocampal neurons (Cohen and Nadler, 1997) and increased concentrations of L-proline activate NMDA and AMPA receptors directly (Henzi et al., 1992; Martin et al., 1992; Pace et al., 1992). Accordingly, elevated brain L-proline levels in PRODH deficient animals were shown to affect neuronal function and lead to a neurological phenotype (Gogos et al., 1999; Paterlini et al., 2005). Together, these findings suggest the need for an accurate regulation of L-proline concentrations in the CNS by specific transport systems. PROT (*Slc6A7*) is brain-specific and presynaptically localized in subsets of glutamatergic nerve terminals with partial apposition to AMPA and NMDA receptors (Crump et al., 1999; Renick et al., 1999), suggesting a role in precisely controlling L-proline levels at these synapses. Additionally, the members of the amino acid transport system A SNAT1 (*Slc38A1*) and SNAT2 (*Slc38A2*) are expressed both in glutamatergic neurons and glial cells and regulate the intracellular pool of L-proline (Mackenzie et al., 2003; González-González et al., 2005). B0AT2/v7–3/NTT7 (*Slc6A15*) and B0AT3/NTT4/XT1 (*Slc6A17*), two members of the neutral amino acid transport family, are also expressed in neurons and facilitate the uptake of L-proline when heterologously expressed (Bröer et al., 2006; Parra et al., 2008). The existence of such a variety of transport-systems for L-proline compartmentalization suggest a strong biological requirement for the regulation of local concentrations of this amino acid in brain, but leaves broad uncertainties about the contribution of the transport mediated by PROT as well as its physiological importance at glutamatergic synapses. Our data show that L-proline uptake was reduced by more than 70% in PROT KO mice, indicating that PROT is the most prominent proline transporter in the analyzed brain regions. Furthermore, they suggest that no functionally compensatory changes in other L-proline transporters arose as a result of PROT inactivation.

The changes in glutamatergic transmission triggered by increased extracellular L-proline levels in brains of PRODH-deficient animals (Paterlini et al., 2005) lead to behavioral and molecular alterations that would be expected to occur similarly in PROT KO mice. In line with the observation that *Drosophila* (Hayward et al., 1993) and mice (Gogos et al., 1999) deficient for PRODH display movement abnormalities and impaired locomotor activity, PROT KO mice showed comparable hypoactivity. Likewise, CaMKIIα, which is downregulated in cortex of PRODH-deficient mice (Paterlini et al., 2005), was also reduced in synaptosomes prepared from PROT^−/−^ mice. Consistent with the regulation of excitatory neurotransmission mediated by L-proline (Henzi et al., 1992; Martin et al., 1992; Pace et al., 1992; Cohen and Nadler, 1997), and the role of neurotransmitter transporters modulating the expression of synaptic components in their respective synapses (Kristensen et al., 2011), we also observed decreased amounts of selective subunits of ionotropic glutamate receptors in the synaptosomal fractions from PROT KO animals. The AMPA receptor GluA1 subunit is critical for activity-dependent postsynaptic strengthening of excitatory synapses and its downregulation is a crucial homeostatic protective mechanism directed to avoid abnormal overexcitation, thus resetting the synaptic activity to basal levels (O’Brien et al., 1998; Yuen et al., 2007). By using cultured cortical neurons, we found that inhibition of PROT function by GGFL led to reduced expression of GluA1. This ruled out that developmental changes resulting from PROT deficiency are causal for alterations in glutamatergic synapse biochemistry, and supported a direct effect of PROT activity on GluA1 expression and/or stability. It should also be noted that GluA1 is inserted into the synaptic membrane in a CaMKII-dependent process (Hayashi et al., 2000). The reduction in CaMKIIα levels might also contribute to the decrease of GluA1 abundance by increasing the intracellular levels of GluA1 that could then be sorted for degradation by different molecular machinery (Ehlers, 2000; Patrick et al., 2003). Furthermore, GluA1 expression also influences CaMKIIα abundance, as demonstrated by the reduction in CaMKIIα mRNA and protein levels in GluA1 KO animals (Zhou et al., 2009). It is worthy of note that a similar GluA1 reduction in neurons has also been reported upon inactivation of the glutamate transporter EAAT3 (Jarzylo and Man, 2012) and the neutral amino acid transporter, capable of L-proline uptake, *Slc6A15* (Santarelli et al., 2016). Together with our data on PROT inactivation, these findings point to a significant role of specific neuronal transporters in regulating excitatory synapses by influencing the number of AMPA receptors. It could be envisaged that such regulation would be particularly important in those synapses that are not surrounded by glial processes expressing neurotransmitter transporters.

PROT inactivation does not lead to amnesia, altered L-glutamate uptake and neurotoxicity, which are induced upon administration of larger amounts of L-proline (Wyse and Netto, 2011), thus discarding the possibility of local extreme extracellular concentrations of L-proline in brains of PROT KO mice. Furthermore, PROT mutants exhibit neither a statistically significant disruption in PPI nor increased abundance of COMT in cerebral cortex, as observed in PRODH-deficient animals. Therefore, our study reveals that PROT inactivation does not trigger the full range of symptoms previously identified in animal models of metabolic hyperprolinemia, suggesting that several symptoms observed in this syndrome are directly linked to the loss of PRODH. Interestingly, a similar finding has been reported in mice and human patients lacking functional glycine transporter 1 (GLYT1, *Slc6A9*). GLYT1-deficiency leads to some, but not the full range, of the symptoms seen in glycine encephalopathy, an inherited human disease triggered by a primary defect in the glycine cleavage system that degrades excess of intracellular glycine, which leads to elevated levels of glycine in the blood and cerebrospinal fluid of the affected individuals (Gomeza et al., 2003; Kurolap et al., 2016).

A surprising result obtained here is that inactivation of the PROT gene does not lead to widespread adaptive alterations of synapse biochemistry in forebrain regions where PROT is expressed. Whereas our Western blot analyses indicate similar expression levels of different glutamate receptor subunits and CaMKIIα in the hippocampus of all genotypes analyzed, a significant downregulation of specific components of the respective synapses occur in the thalamus and cortex in response to inactivation of the PROT gene. This dissimilar expression pattern of synaptic proteins could be as a result of unequal levels of PROT in specific brain regions. However, PROT is widely expressed throughout the CNS, with the highest levels in cortex, hippocampus and thalamus (Velaz-Faircloth et al., 1995), resulting in prominent synaptosomal high affinity L-proline uptake (Hauptmann et al., 1983). Our data suggest that the lack of PROT results in restricted compensatory mechanisms depending on the brain region.

Previous studies have shown that a reduction of GluA1 and CaMKIIα expression is often associated with altered learning acquisition and memory behavior. However, both cognitive functions were unaffected by loss of PROT. Memory extinction, a process necessary for behavioral flexibility, has also been shown to depend on glutamatergic neurotransmission (Parnaudeau et al., 2015). Memory extinction is facilitated by enhancing AMPA receptor activity in cortex (Zushida et al., 2007) and by increasing AMPA receptor expression in the infralimbic cortex (Wang et al., 2013). Conversely, reduced levels of synaptic AMPA receptors (Wang et al., 2013) and deficient CaMKIIα function (Kimura et al., 2008) interfere with memory extinction. Thus, the behavioral deficits observed in PROT KO mice might result from altered glutamatergic neurotransmission within the cortex. Interestingly, also the thalamus has been shown to play a vital role in a wide range of cognitive functions, among them the regulation of attentional and emotional behaviors, most likely due to its role as nodal point integrating information from different brain regions (Kirouac, 2015; Ouhaz et al., 2015). Therefore, our data suggest that the observed changes in the expression levels of GluA1 and CaMKIIα triggered by the absence of PROT both in cortex and thalamus could significantly contribute to the deficits in memory extinction observed in PROT deficient animals. Clearly, future studies analyzing the direct consequences of reduced PROT activity on glutamatergic synaptic transmission in both brain regions are required to unravel the effects of altered extracellular L-proline concentrations on both cellular and systemic level.

The results presented in this study also reveal a previously unrecognized role for PROT in regulating emotional behaviors to novelty. The decreased explorative behavior that PROT deficient mice showed in the central area of the open field arena was not caused by increased anxiety, since PROT^−/−^ animals showed unaltered performance in two different tests that identify anxiety-like behavior. This finding is in line with the observation that hyperprolinemia does not induce anxiety in PRODH deficient mice (Gogos et al., 1999). The decreased approach-behavior observed in PROT KO animals when confronted with a new stimulus in a familiar environmental context uncovers a significant role of PROT in novelty exploration, a behavior needed to respond to environmental changes and, consequently, essential for animal survival (Dulawa et al., 1999; Wiedholz et al., 2008; Meirsman et al., 2016). It is worthy of note that motivation toward novel objects in mice has been related with novelty-seeking in humans, a behavior whose altered intensification has been associated with mental disorders and violent conduct (Kim et al., 2005).

Altogether, deficiencies observed in PROT^−/−^ mice both in novelty-seeking and memory extinction point to a role of PROT in modulating behaviors that allow a different response choice when task contingencies change. Such behaviors are known to be altered in several psychiatric and neurologic disorders. Thus, exploration to novel stimuli is reduced in individuals with autism, Parkinson’s and Alzheimer’s disease (Powell et al., 2004), and cognitive flexibility is decreased in schizophrenia and autism (D’Cruz et al., 2011). Notably, high levels of L-proline in cerebrospinal fluid have been associated with schizophrenia (Luykx et al., 2015) and transcriptomic analyses have revealed a decreased expression of PROT in post-mortem autistic brains (Voineagu et al., 2011). It could be envisaged that some behavioral symptoms observed in human brain disorders may be attributable to PROT malfunction. In this context, PROT might be considered as candidate disease gene leading to intermediate phenotypes, or endophenotypes, in multifactorial mental disorders, and consequently, PROT^−/−^ mice might further constitute a new useful animal model to study one particular dimension or endophenotype of human psychiatric disorders. Clearly, genome-wide association studies are required to analyze the potential link of PROT and vulnerability to psychiatric diseases in the future.

## Author Contributions

DS generated the knockout mice and performed initial biochemical, molecular and behavioral analyses. JM performed Western blot analyses, prepared and analyzed neuronal cell cultures and supported behavioral studies. SS performed behavioral experiments and data analysis. VE designed, performed and analyzed behavioral studies and reviewed the final manuscript. JG designed the study, secured funding, provided expertise, analyzed data, prepared figures of the manuscript, wrote the original draft and reviewed and edited the final manuscript.

## Conflict of Interest Statement

The authors declare that the research was conducted in the absence of any commercial or financial relationships that could be construed as a potential conflict of interest.
